# Combined application of chondroitinase ABC and photobiomodulation with low-intensity laser on the anal sphincter repair in rabbit

**DOI:** 10.1186/s12876-021-02047-2

**Published:** 2021-12-15

**Authors:** Arash Sarveazad, Abazar Yari, Arash Babaei-Ghazani, Marjan Mokhtare, Mansour Bahardoust, Siavash Asar, Jebreil Shamseddin, Mahmoud Yousefifard, Asrin Babahajian

**Affiliations:** 1 Nursing Care Research Center, Iran University of Medical Sciences, Tehran, Iran; 2 Colorectal Research Center, Iran University of Medical Sciences, Tehran, Iran; 3 Department of Anatomy, Faculty of Medicine, Alborz University of Medical Sciences, Karaj, Iran; 4 Dietary Supplements and Probiotics Research Center, Alborz University of Medical Sciences, Karaj, Iran; 5 Neuromusculoskeletal Research Center, Department of Physical Medicine and Rehabilitation, Iran University of Medical Sciences, Tehran, Iran; 6 Department of Physical Medicine and Rehabilitation, University of Montreal Health Center, Montreal, Canada; 7 Department of Epidemiology, School of Public Health, Shahid Beheshti University of Medical Sciences, Tehran, Iran; 8 Department of Anesthesiology, Kerman University of Medical Sciences, Kerman, Iran; 9 Infectious and Tropical Diseases Research Center, Hormozgan Health Institute, Hormozgan University of Medical Sciences, Bandar Abbas, Iran; 10 Physiology Research Center, Iran University of Medical Sciences, Hemmat Highway, P.O Box: 14665-354, Tehran, Iran; 11 Liver and Digestive Research Center, Research Institute for Health Development, Kurdistan University of Medical Sciences, P.O Box: 14665-354, Sanandaj, Iran

**Keywords:** Chondroitin ABC Lyase, Photobiomodulation, Low-intensity laser, Rabbit, Fecal incontinence

## Abstract

**Background:**

Photobiomodulation with low-intensity laser (LIL) and chondroitinase ABC (ChABC) can repair damaged muscle tissue, so the aim of this study was to investigate the effect of co-administration of these two factors on anal sphincter repair in rabbits.

**Methods:**

Male rabbits were studied in 5 groups (n = 7): Control (intact), sphincterotomy, laser, ChABC and laser + ChABC. 90 days after intervention were evaluated resting and maximum squeeze pressures, number of motor units, collagen amount, markers of muscle regeneration and angiogenesis.

**Results:**

Resting pressure in the Laser + ChABC group was higher than the sphincterotomy, laser and ChABC groups (*p* < 0.0001). Maximum squeeze pressure in the all study groups was higher than sphincterotomy group (*p* < 0.0001). In the laser + ChABC and ChABC groups, motor unit numbers were more than the sphincterotomy group (*p* < 0.0001). Collagen content was significantly decreased in the laser (*p* < 0.0001) and laser + ChABC groups. ACTA1 (*p* = 0.001) and MHC (*p* < 0.0001) gene expression in the Laser + ChABC group were more than the laser or ChABC alone. VEGFA (*p* = 0.009) and Ki67 mRNA expression (*p* = 0.01) in the Laser + ChABC group were more than the laser group, But vimentin mRNA expression (*p* < 0.0001) was less than the laser group.

**Conclusion:**

Co-administration of ChABCs and photobiomodulation with LIL appears to improve the tissue structure and function of the anal sphincter in rabbits more than when used alone.

**Supplementary Information:**

The online version contains supplementary material available at 10.1186/s12876-021-02047-2.

## Background

Anal sphincter injury is one of the pelvic floor disorders, a major cause of fecal incontinence (FI). FI occurs in patients due to the injured anal sphincter's weakness in obstruction of the anal canal [1, 2] irrespective of age, gender, and social status, and its prevalence is 2–15% [3, 4]. FI severely affects people's quality of life in social embarrassment, social activities, and occupational disorder [5]. The main treatment for FI is various surgeries such as injection of bulking agents [6], tibial nerve stimulation [7], neuromodulation [8], sacral nerve stimulation [9], and cecostomy [9]. These treatments do not have long-lasting and effective results. Therefore, research is performing to replace permanent treatments such as artificial sphincters [10] and the application of stem cell therapy [11–13]. Many studies have shown a low-intensity laser (LIL) role in the repair and remodeling of muscle tissue [14–16]. Therefore, one of the interventions that can be used as an effective treatment in repairing damaged sphincter muscle tissue is photobiomodulation with LIL. Silent satellite cells beneath the basement membrane of muscle fibers play an essential role in muscle regeneration. LIL can be waking up these satellite cells and entering the proliferative phase [17–19].

On the other hand, fibroblasts have a wide range of chromophores stimulated by different wavelengths of laser [20]. Following stimulation of fibroblasts, several growth factors such as FGF beta IGF-1 are secreted from these cells, essential for tissue repair and the formation of new vessels [21]. Another intervention that can repair the damaged sphincter muscle tissue involves reducing the amount of chondroitin sulfate (CS) in the damaged tissue. CS is a substance widely found in the extracellular matrix and cell surface in the form of chondroitin sulfate proteoglycan (CSPG) [22]. It reduced the amount of CS naturally required for the myofibrils joining process and is essential for the construction of functional cellular syncytium such as skeletal muscle [23–25]. Chondroitin ABC Lyase (ChABC) is a bacterial enzyme that can induce CSPG digestion and decrease CS in the extracellular matrix [26, 27]. Given the ability of LIL to awaken silent satellite cells and the ability of ChABC to reduce CS to organize these awakened satellite cells into a functional muscular syncytium, co-administration of these two therapies can be an effective and permanent approach to muscle repair. Therefore, in this study, we aimed to investigate the combined effect of ChABC and photobiomodulation with LIL for the repair of the injured anal sphincter in rabbits.

## Methods

### Study design

The present experimental study was designed and performed according to the ARRIVE standard guidelines (Animal Research Reporting of in Vivo Experiments) Guidelines. In this study, 35 male rabbits with 5 groups (n = 7) were studied. To minimize bias in the study, the animals were randomly divided into study groups. The researchers performing the experimental, data gathering, and interpretation of the data were blinded to the study. Each rabbit was kept in separate cages during the study procedure.

### Animals, housing and husbandry

In this study, 35 healthy New Zealand white male rabbits weighing 2.5–3 kg were prepared from Pasteur Institute of Iran, Iran, Tehran. All animals were individually housed in rabbit cages (grid floor, standard water container, and feed) in the standard conditions (21 ± 3 °C and 12-h light and dark cycle) by access to the sufficient storage of fresh water and food.

### Allocating animals to experimental groups

Animals randomly were divided to 5 equal groups (n = 7) consist of: (A) Control, (B) Sphinctrotomy, (C) Laser, (D) ChABC, and (E) ChABC + Laser (Table 1).

**Table 1:** Experimental groups of study

Experimental groups	Procedure
Control	Intact without intervention
Sphinctrotomy	Sphinctrotomy + Normal saline (in situ injection)
Laser	Sphinctrotomy + Laser irradiation (90 s/Daily/up to 14 days)
ChABC	Sphinctrotomy + 10 μl of 100 U/ml ChABC (in situ injection)
ChABC + Laser	Sphinctrotomy + 10 μl of 100 U/ml ChABC + Laser irradiation (90 s/Daily/up to 14 days)

ChABC: Chondroitinase ABC

### Sphincterotomy

Sphincterotomy was performed based on the fourth degree rupture of the 1999 Sultan classification [external anal sphincter (EAS), internal anal sphincter (IAS), and anal mucosa tear] [28]. As in our previous study [12], after anesthesia with ketamine (80 mg/kg) and xylazine (10 mg/kg) in all rabbits, sphincterotomy in lithotomy position after disinfection of anus and perineal area of the left side of sphincter was performed at a depth of 1 cm.

### Photobiomodulation with LIL

In this study, a CW diode laser with a wavelength of 660 nm and power of 100 mW (model Heltschl, model ME-TL10000-SK) was used. The laser irradiation device was mounted on a metal height-adjustable stand (2 cm to the lesion site) to stabilize the laser's irradiation point. The location of the laser irradiation was three points (each point 5 mm wide). The first and second points included the border between the lesion and the healthy tissue of the sphincter (both sides of the lesion) and the third point was the center of the lesion. The irradiation time at each point was 30 s (total of three points 90 s).Immediately after sphincterotomy, the lesion site was irradiated. Laser irradiation was performed every day for 14 days at the specified time (2 pm).

### ChABC preparation and injection

ChABC (Sigma, C3667) diluted with 0.01% bovine serum albumin in normal saline (10 µl of 100U/ml/in situ). A single dose was injected immediately after sphincterotomy with a Hamilton syringe attached to the microinjection device. The injection was made by inserting a 5 mm needle on two edges of the lesion in the sphincter (5 µl per edge) slowly over 2 min. After an injection to prevent ChABC leakage, the needle remained in place for 2 min.

### Outcomes

#### Measurement of resting and maximum squeeze pressures (Manometry)

The standard anorectal catheter (4.7 mm 8 h) and a pressure transducer (145 Trades Blvd. East, Unit #34, Mississauga, ON, L4Z 3L3, Canada) were used to perform manometry. After three months, to measure the pressure of the anal sphincter, after inserting the probe into the rectum and positioning the balloon baseline pressure, at the same time as the probe was pulled out (constant rate of 0.05 cm/s), the sphincter pressure profile (resting and maximum squeeze pressures) was recorded. Manometry was done three times for each rabbit by the blind operator.

#### Electromyography (EMG)

To record EMG from the sphincterotomy site after three months, a Synergy on Nicolet EDX System (Natus Medical Corporate USA) was used with disposable electrode and needle (Gauge 30, diameter 0.3 mm, length 25 mm, and recording area of 0.02 mm^2^/Ambu Copenhagen, Denmark). Rabbits were placed in a lithotomy position (without anesthesia or relaxant). The EMG needle was inserted vertically at the sphincterotomy site (in the boundary of anus mucosa and skin/depth of 0.5 cm). After setting the device (sweep: 10 ms/cm and Sensitivity: 100–200), the number of motor units was recorded and counted for 20 s for each rabbit by the blind operator.

#### Histological assay

Ninetydays after ChABC injection and the first laser irradiation, animals (n = 3) were anesthetized. After cardiac perfusion (4% paraformaldehyde solution), the anal sphincter was completely removed and post-fixed (4% paraformaldehyde solution overnight) in each group. After tissue preparation and paraffin molding process, serial transverse sections were prepared (10 µm thickness). The rest of the animals in each group (n = 4) after deep anesthesia were sacrificed, and their anal sphincter was freshly removed. Finally, the sphincterotomy site was separated and stored at − 80 °C for Real-time PCR assessment.

#### Mallory's Trichrome staining and quantitative assay of collagen

In each group, three animals were selected, and three sections from each animal were stained with Mallory's Trichrome method for collagen assessment. In this method, collagen fibers are seen in blue. After photographing the sphincterotomy site (light microscopy ×40), the area occupied by blue dye to the sphincterotomy site was considered to the amount of collagen [11]. The ratio was calculated by ImageJ/Fiji 1.46 software.

#### Immunohistochemistry (IHC)

The list of primary and secondary antibodies and their number codes are given in Table S1. IHC was performed according to standard protocols. Briefly as follows: First incubation of tissue sections was done in 95 °C with citrate buffer (PH = 6) for 10 min (due to antigen retrieval). After washing with PBS, second incubation was done by H_2_O_2_ 10% (diluted in methanol) for 15 min. Third incubation was done at 37 °C by Goat Serum 10% (Sigma, USA) for 30 min (due to the blocking process). The primary antibody did fourth incubation at four °C, humidified environment for 24 h. After three times washing with PBS, final incubation was done by the secondary antibody in the dark, 37 °C, humidified atmosphere for 2 h. After the exact final three times washing with PBS, DAPI staining was done due to cell nuclei staining. Tissue sections were assessed and photographed (fluorescence microscopy × 10).

#### RNA extraction, cDNA synthesis, and real-time PCR

The total RNA of Glyceraldehyde-3-phosphate dehydrogenase (GAPDH), Vascular endothelial growth factors A (VEGFA), Myosin heavy chain (MYH), Actin alpha 1, skeletal muscle (ACTA1), Ki67, and Vimentin was extracted by EURx RNA Extraction Kit. Then, 1 mg of total RNA was reverse transcribed using EURx cDNA synthesis kit and random hexamer primer. Real-time PCR was performed using Stepone ABI (Thermo fisher scientific, USA), QuantiTect primers (Kyagen Company), and Master Mix Cyber Green PCR. The sequence of primers is shown in Table S2. Data were normalized to GAPDH as a housekeeping gene.

#### Statistical methods

Quantitative data are reported as mean (standard deviation) or median (range) and qualitative data as frequency (percent). One-way ANOVA and Bonferroni post hoc was used to assess any differences in studied groups. A *P*-value less than 0.05 was considered significant. SPSS® for windows version 16 software was used for analysis.

## Results

### Resting pressure and maximum squeeze pressures

90-day after sphincterotomy, a significant decrease was observed in resting pressure in all treated groups compared to control animals (*df*: 4, 34; F = 226.6; *p* < 0.0001). Photobiomodulation with LIL (*p* < 0.0001) and administration of ChABC (*p* < 0.0001) led to a significant improvement in resting pressure compared to the sphincterotomy group. Co-application of ChABC + Laser increased the resting pressure compared to laser and ChABC alone (*p* < 0.0001). There is no significant difference between the laser and ChABC group (*p* > 0.99).

**Fig. 1:**
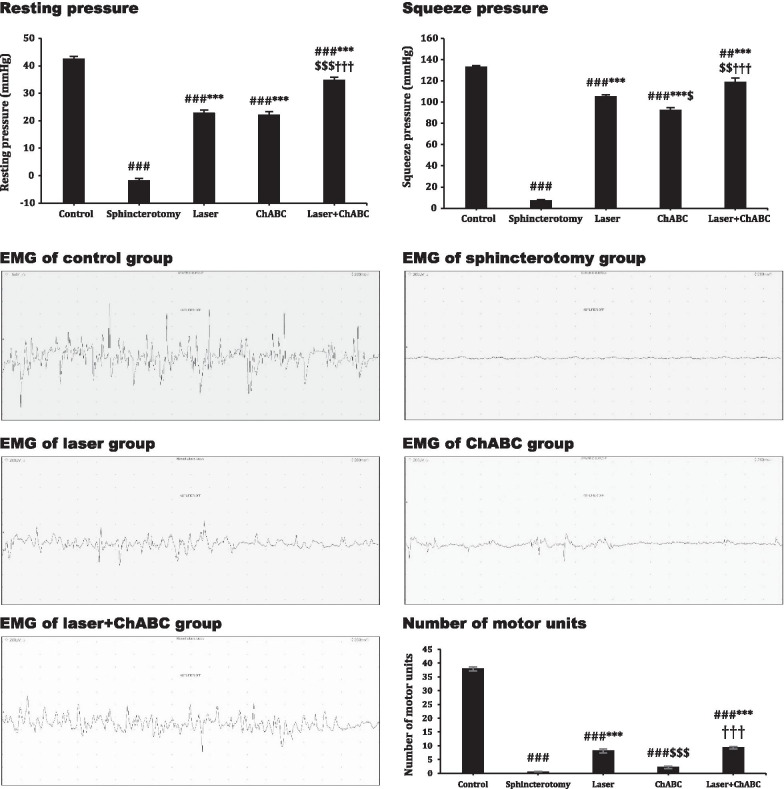
Effect of laser, chondroetinase ABC (ChABC) and combination of laser and ChABC (Laser + ChABC) on monometery parameters and electromyography (EMG) of anal sphincter. Data are presented as means ± SEM (n = 7 animal per group). ***, represent significant level at *p* < 0.0001 with sphincterotomy group. ###, represent significant level at *p* < 0.0001 with control group (intact animals). ##, represent significant level at *p* < 0.01 with control group. $$$, represent significant level at *p* < 0.0001 with laser group. $$, represent significant level at *p* < 0.01 with laser group $, represent significant level at *p* < 0.05 with laser group. †††, represent significant level at *p* < 0.0001 with ChABC group

The maximum squeeze pressure in the sphincterotomy group was significantly lower than other groups (*df*: 4, 34; F = 395.5; *p* < 0.0001). Treatment with laser, ChABC, and ChABC + Laser led to a significant improvement in the maximum squeeze pressure compared to sphincterotomized animals (*p* < 0.0001). The laser had better efficacy on maximum squeeze pressure than ChABC (*p* = 0.011). Co-administration of ChABC + Laser is associated with higher maximum squeeze pressure than laser (*p* = 0.005) and ChABC (*p* < 0.0001) alone (Fig. 1).

### Motor unit numbers

Ninety days after interventions, the number of motor units in all treated animals was significantly lower than intact animals (*df*: 4, 34; F = 635.6; *p* < 0.0001). The laser (*p* < 0.0001) and ChABC + Laser groups (*p* < 0.0001) displayed substantially more motor units than the sphincterotomy group (*p* < 0.0001). However, administration of ChABC did not affect the number of motor units (*p* = 0.567). Finally, there is no significant difference between laser alone and ChABC + Laser in increasing motor units (*p* > 0.99) (Fig. 1).

### Collagen and muscle content in the injured sphincter

**Fig. 2:**
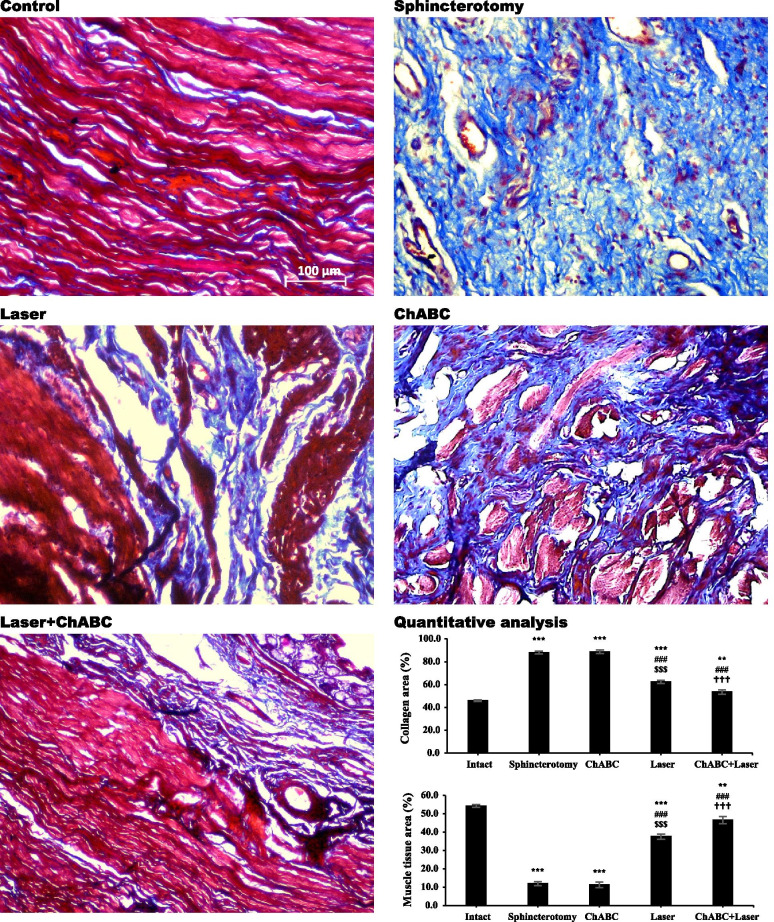
Percentage of collagen and muscle tissues in sphincterotomy site of anal sphincter after laser, Chondroetinase ABC (ChABC) and combination of ChABC and laser (ChABC + Laser) treatments. Data are presented as means ± SEM (n = 3 animal per group). ***, represent significant level at *p* < 0.0001 with intact group. **, represent significant level at *p* < 0.01 with intact group. ###, represent significant level at *p* < 0.0001 with sphincterotomy group. $$$, represent significant level at *p* < 0.0001 with ChABC group. †††, represent significant level at *p* < 0.0001 with laser group. Mallory's trichrome staining, anal sphincter, rabbit (× 20)

After sphincterotomy, the collagen content of the anal sphincter was increased (*df*: 4, 11; F = 206.9; *p* < 0.0001). Photobiomodulation with LIL (*p* < 0.0001) and a combination of ChABC + Laser (*p* < 0.0001) decreased the collagen amount compared to the sphincterotomy group. However, administration of ChABC did not reduce the collagen content (*p* > 0.99). Similar results were seen in the muscle tissue (*df*: 4, 11; F = 207.3; *p* < 0.0001) (Fig. 2).

### α-actin expression

**Fig. 3:**
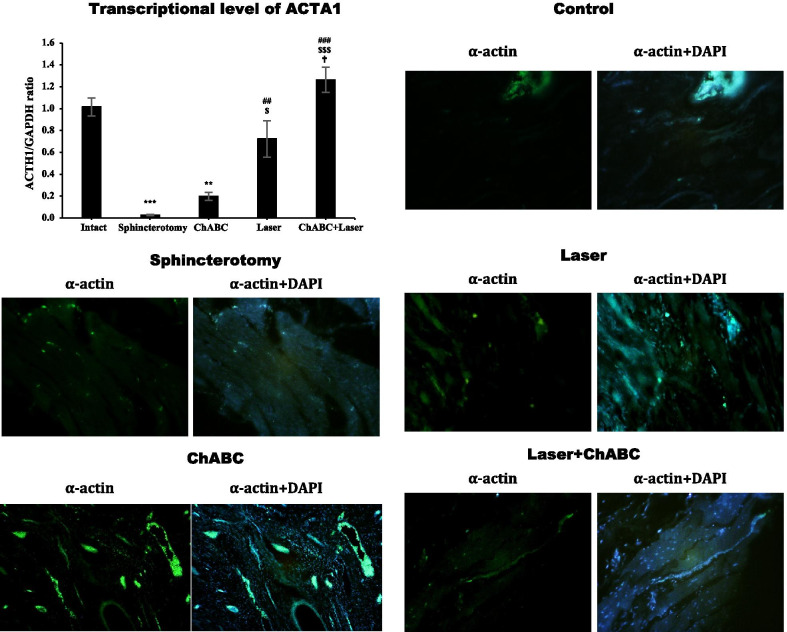
Gene and protein expression of α-actin in injury site of external anal sphincter after laser, chondroitinase ABC (ChABC) and combination of laser and ChABC (Laser + ChABC) treatments. α-actin expression are observed in laser and ChABC groups (green). ***, represent significant level at *p* < 0.0001 with intact group. **, represent significant level at *p* < 0.01 with intact group. ###, represent significant level at *p* < 0.0001 with sphincterotomy group. ##, represent significant level at *p* < 0.01 with sphincterotomy group. $$$, represent significant level at *p* < 0.0001 with ChABC group. $, represent significant level at *p* < 0.05 with ChABC group. †, represent significant level at *p* < 0.05 with laser group. Data are presented as means ± SEM (n = 3 animal per group). Real-time PCR and immunohistochemistry of external anal sphincter, rabbit (× 10)

Gene expression of α-actin in the injury site of the external anal sphincter after sphincterotomy was significantly reduced (*df*: 4, 10; F = 27.9; *p* < 0.0001). Photobiomodulation with LIL (*p* = 0.005) and ChABC + Laser (*p* < 0.0001) significantly increased the α-actin gene expression and reached to intact animals (*p*_for laser_ = 0.643 and *p*_for ChABC + Laser_ > 0.99). ChABC administration did not change the level of α-actin gene expression (*p* > 0.99). The level of α-actin gene expression in the ChABC + Laser group was significantly higher than ChABC (*p* < 0.0001) and Laser (*p* = 0.033) alone treated animals. The immunohistochemical assessment showed similar findings (Fig. 3).

### Myosin heavy chain expression

**Fig. 4:**
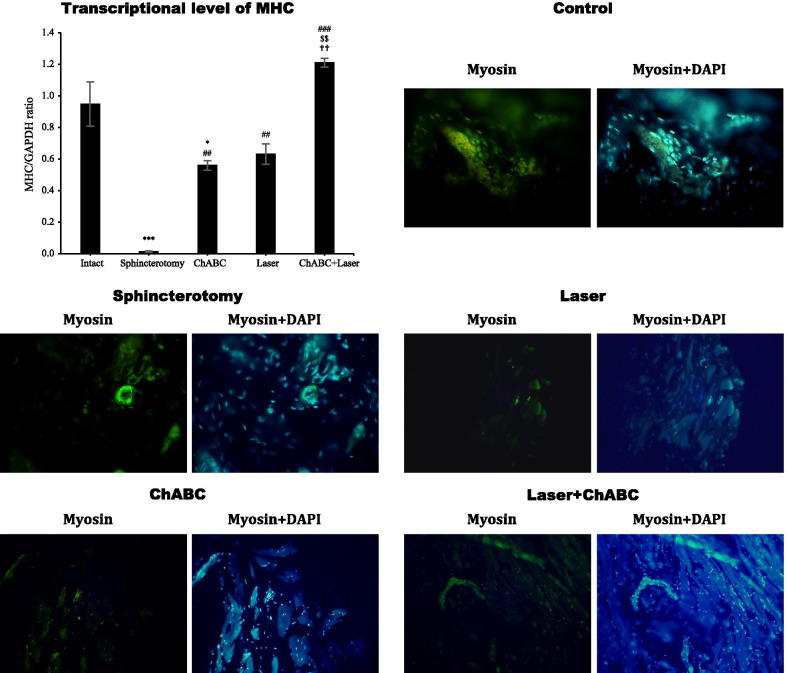
Gene and protein expression of myosin (MHC) in injury site of external anal sphincter after laser, chondroitinase ABC (ChABC) and combination of laser and ChABC (Laser + ChABC) treatments. Myosin expression are observed in laser and ChABC groups (green). ***, represent significant level at *p* < 0.0001 with intact group. *, represent significant level at *p* < 0.05 with intact group. ###, represent significant level at *p* < 0.0001 with sphincterotomy group. ##, represent significant level at *p* < 0.01 with sphincterotomy group. $$, represent significant level at *p* < 0.01 with ChABC group. ††, represent significant level at *p* < 0.01 with laser group. Data are presented as means ± SEM (n = 3 animal per group). Real-time PCR and immunohistochemistry of external anal sphincter, rabbit (× 10)

Gene expression of myosin heavy chain in the external anal sphincter's injury site after sphincterotomy was significantly reduced (*df*: 4, 10; F = 39.4; *p* < 0.0001). ChABC (*p* = 0.003), photobiomodulation with LIL (*p* = 0.001) and ChABC + Laser (*p* < 0.0001) administration significantly increased the myosin heavy chain gene expression. The level of myosin heavy chain gene expression in the ChABC + Laser group was significantly higher than ChABC (*p* = 0.001) and laser (*p* = 0.002) alone treated animals. The immunohistochemical assessment showed similar findings (Fig. 4).

### VEGF heavy chain expression

**Fig. 5:**
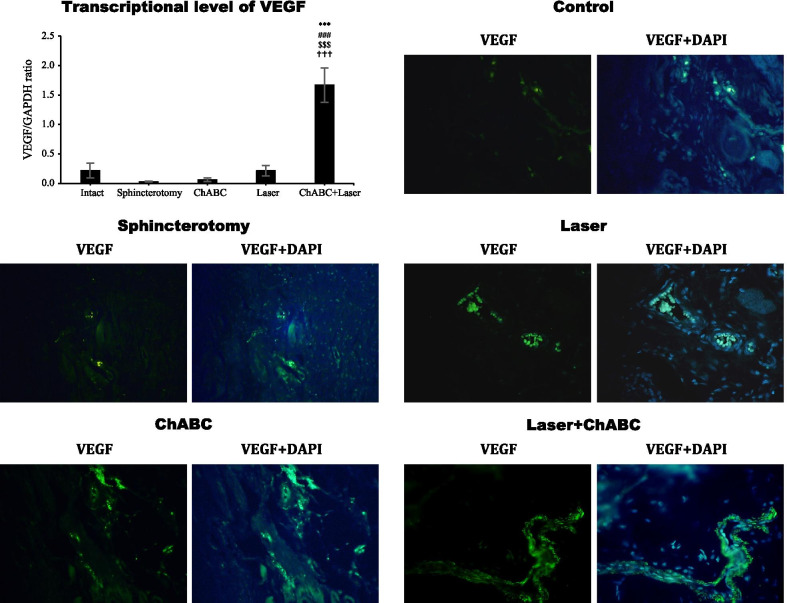
Gene and protein expression of vascular endothelial growth factor (VEGF) in injury site of external anal sphincter after laser, chondroitinase ABC (ChABC) and combination of laser and ChABC (Laser + ChABC) treatments. VEGF expression are observed in laser and ChABC groups (green). ***, represent significant level at *p* < 0.0001 with intact group. ###, represent significant level at *p* < 0.0001 with sphincterotomy group. $$$, represent significant level at *p* < 0.0001 with ChABC group. †††, represent significant level at *p* < 0.0001 with laser group. Data are presented as means ± SEM (n = 3 animal per group). Real-time PCR and immunohistochemistry of external anal sphincter, rabbit (×10)

Gene expression of VEGF in the injury site of external anal did not change after sphincterotomy (*p* > 0.99). Although, ChABC (*p* > 0.99) and laser (*p* > 0.99) administration did not change the VEGF gene expression but the combination therapy (ChABC + Laser) significantly increased level of VEGF expression (*df*: 4, 10; F = 22.0; *p* < 0.0001). The VEGF expression level was significantly higher in the ChABC + Laser group than laser and ChABC alone treated animals (*p* < 0.0001). The immunohistochemical assessment showed similar findings (Fig. 5).

### Vementin expression

**Fig. 6:**
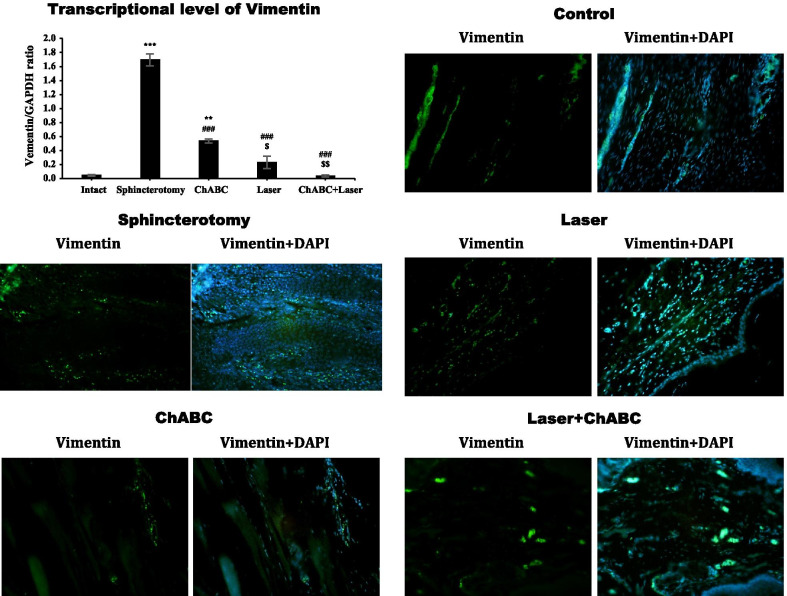
Gene and protein expression of vementin in injury site of external anal sphincter after laser, chondroitinase ABC (ChABC) and combination of laser and ChABC (Laser + ChABC) treatments. Vimentin expression are observed in laser and ChABC groups (green). ***, represent significant level at *p* < 0.0001 with intact group. **, represent significant level at *p* < 0.01 with intact group. ###, represent significant level at *p* < 0.0001 with sphincterotomy group. $$, represent significant level at *p* < 0.01 with ChABC group. $, represent significant level at *p* < 0.05 with ChABC group. Data are presented as means ± SEM (*n* = 3 animal per group). Real-time PCR and immunohistochemistry of external anal sphincter, rabbit (× 10)

Gene expression of vementin in the injury site of the external anal sphincter after sphincterotomy was significantly increased (*df*: 4, 10; F = 147.2; *p* < 0.0001). ChABC administration (*p* < 0.0001), photobiomodulation with LIL (*p* < 0.0001) and ChABC + Laser (*p* < 0.0001) administration significantly decreased the vementin gene expression. The level of vimentin expression reached intact animals in laser (*p* = 0.464) and ChABC + Laser (*p* > 0.99) groups. The vimentin gene expression level in laser (*p* = 0.035) and ChABC + Laser (*p* = 0.001) was less than the ChABC group. The immunohistochemical assessment showed similar findings (Fig. 6).

### Ki67 expression

**Fig. 7:**
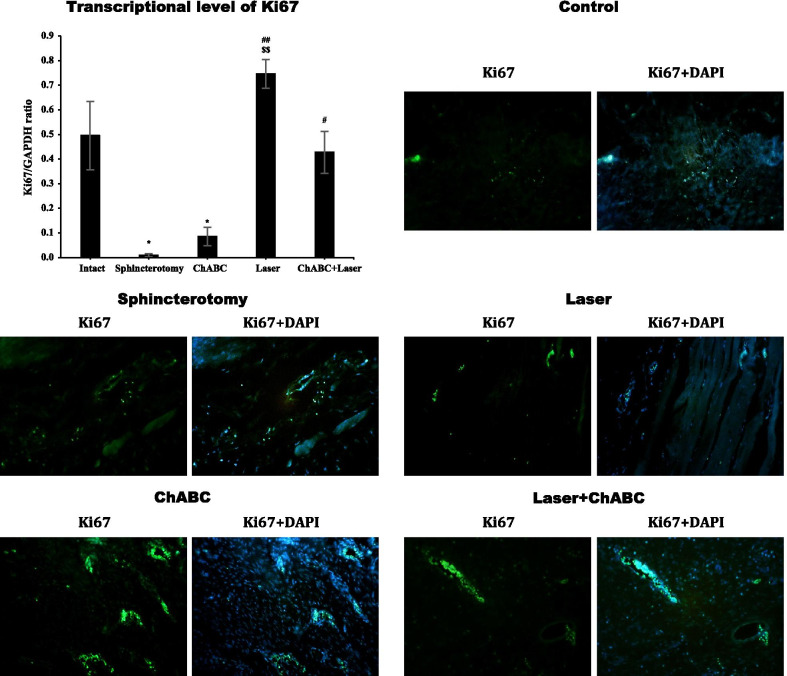
Gene and protein expression of Ki67 in injury site of external anal sphincter after laser, Chondroitinase ABC (ChABC) and combination of laser and ChABC (Laser + ChABC) treatments. Ki67 expression are observed in laser and ChABC groups (green). *, represent significant level at *p* < 0.05 with intact group. ##, represent significant level at *p* < 0.01 with sphincterotomy group. #, represent significant level at *p* < 0.05 with sphincterotomy group. $$, represent significant level at *p* < 0.01 with ChABC group. Data are presented as means ± SEM (n = 3 animal per group). Real-time PCR and immunohistochemistry of external anal sphincter, rabbit (× 10)

Gene expression of Ki67 in the injury site of the external anal sphincter after sphincterotomy was significantly decreased (*df*: 4, 10; F = 14.6; *p* < 0.0001). Photobiomodulation with LIL (*p* = 00.1) and ChABC + Laser (*p* = 0.04) administration significantly increased the Ki67 gene expression. The level of Ki67 expression reached intact animals in laser (*p* = 0.501) and ChABC + Laser (*p* > 0.99) groups. Ki67 expression was significantly higher in the laser group compared to the ChABC group (*p* = 0.002). The immunohistochemical assessment showed similar findings (Fig. 7).

## Discussion

Given the ability of ChABC and photobiomodulation with LIL in muscle regeneration, the present study aimed to investigate the effect of co-administration of laser and ChABC in the repair of the anal sphincter to provide a novel treatment strategy for FI. Injury to the anal sphincter usually results from vaginal delivery, accidents, and explosions, which cause extensive trauma and acute conditions. In such cases, treatment in the early hours after injury is very effective in the final results. Because laser and ChABC administration is possible at any time and does not require a specific process to prepare for use, these treatments are a good option for such acute conditions. Since different laser wavelengths have different abilities to penetrate tissues, the laser wavelength selection in the laser therapy process is important to achieve the maximum therapeutic effect. In our study, damaged tissues (including skin, muscle, and mucosa) are located in the perineal surface area, so a wavelength of 660 nm was chosen for laser therapy. The best laser wavelength in such cases is 600 to 700 nm [29] because, with this wavelength, the best anti-inflammatory effect and secretion of cytokines to the repair process is observed [15, 30–32]. This study's findings show that concomitant use of ChABC and photobiomodulation with LIL improves sphincter function more than when used alone. Manometry findings of sphincterotomized animals significantly were decreased compared to intact animals because of EAS, IAS, and mucosal rupture for induction of sphincterotomy [28]. Anal canal resting pressure is normally due to EAS (15%) and IAS (85%) tone. Maximum squeeze pressure, however, is mainly due to EAS tone [33]. Therefore, it is expected that these two variables to be affected by the damage to the anal sphincter. Ninety days after the prescription of ChABC and the laser irradiation period's completion, resting and maximum squeeze pressures (functional variables) were higher in animals that received these two interventions simultaneously than in other animals. However, there is no trend in tissue variables. The amount of collagen, muscle, and number of motor units in the sphincter of animals that received the laser had the most change (decrease in collagen and increase in muscle and motor units). In the ChABC or combined group, there is no change beyond the laser group. The interpretation of this finding is that ChABC and photobiomodulation with LIL in improving the sphincter's contractile function could have a complementary role. Still, in tissue variables, the determining factor is the laser, and the ChABC has no role. This is to be expected because the role of ChABC in repairing muscle is not to create a new muscle fiber but to connect the muscle fibers to create a functional syncytium [23, 25]. Unlike ChABC, the main role of photobiomodulation with LIL is to create new muscle fibers by stimulating silent satellite cells [19, 34]. Our study results on the expression of Ki67 (indicating proliferation), α-actin, MHC, VEGF, and Vimentin show that the main influential factor in the expression of these proteins is the laser which confirms the effect of LIL on tissue variables compared to ChABC. A significant increase in resting and maximum squeeze pressures in the ChABC group can be due to the connection of small muscle fibers that are endogenously formed at the lesion site without any intervention, as our results show that the amount of muscle in the sphincterotomy group is not zero. It can be concluded that the laser has increased muscle fibers, and on the other hand, ChABC has connected these fibers and created functional syncytium. According to these explanations, the effect of ChABC on functional variables (manometric findings) and the effect of LIL on tissue variables is justifiable. An abundance of mitochondria as photoreceptors in the muscle tissue is a logical assumption for the laser's effect on muscle repair by angiogenesis [18] and stimulating satellite cells to produce new myofibrils [19, 34]. Inconsistent with these statements, our study results also show an increase in ki67 (proliferation factor) and VEGF mRNA expressions in the laser group compared with sphincterotomized animals. In the present study, EMG findings show after sphincterotomy; motor unit numbers decrease significantly compared to intact animals. This finding is not unexpected due to the significant increase in collagen in the sphincterotomy group compared to other groups. Laser and laser + ChABC treatment protocols resulted in a significant increase in motor unit numbers than the sphincterotomy group. ChABC did not affect the number of motor units. In other words, the determinative factor in increment of the number of motor units is laser and consistent with an increase in the amount of muscle in the laser group (rather than the ChABC) compared to the sphincterotomy group. This finding confirms the role of the laser in promoting tissue variables compared to ChABC, which is more effective in improving functional variables. Photobiomodulation with LIL, after muscle injury, causes the contractile myofibrils to increase at the site of injury, thereby promoting muscle function [34, 35]. The laser does this by stimulating the satellite cells adjacent to the adult myofibrils [19]. In addition to increasing myofibrils, laser by modifying the Nuclear Factor Kappa B (NF-κB) expression causes collagen remodeling [36] and reduces collagen levels [37], thereby advancing the muscle repair process. In the present study, the amount of collagen in the laser group also confirms the laser's regulatory role on collagen during the repair process. Unlike laser, ChABC did not play a role in post-lesion collagen modulation. One of the most important muscle repair processes during the first year after injury is the rearrangement of collagen fibers to withstand maximum pressure and traction at the site of injury [38, 39]. The key factor in promoting this process is Vimentin. In addition to collagen remodeling, Vimentin also induces collagen production [38, 40, 41]. On the other hand, the amount of vimentin is inversely related to the number of mature myofibrils formed (the amount of muscle) during the muscle repair process [38]. The amount of Vimentin expression in different groups in the present study confirms these statements. So that, the amount of collagen and muscle in different groups is consistent with the Vimentin expression. In the groups with the highest amount of collagen, the highest Vimentin expression, and the groups with the highest amount of muscle, the lowest Vimentin expression is observed.

## Conclusion

The present study depicted that following the anal sphincter injury, immediate co-application of ChABC and photobiomodulation with LIL improve sphincter function by promoting myogenesis and angiogenesis. This protocol is more efficient than laser and ChABC treatment alone.

## Supplementary Information

**Additional file 1.** **List of antibodies Primer sequences. Table S1:** The list of primary and secondary antibodies. **Table S2:** Primer sequences.

## Data Availability

Datasets of the current study are available from the corresponding authors on reasonable request.

## Abbreviations

ChABC: Chondroitinase ABC
LIL: Low-intensity laser
FI: Fecal incontinence
ACTA: Skeletal muscle alpha-actin
VEGFA: Vascular endothelial growth factor A
CS: Chondroitin sulfate
CSPG: Chondroitin sulfate proteoglycan
GAPDH: Glyceraldehyde-3-phosphate dehydrogenase
VEGFA: Vascular endothelial growth factors A
IHC: Immunohistochemistry
MHC: Myosin heavy chain
