# Supplementary material for: Combined application of chondroitinase ABC and photobiomodulation with low-intensity laser on the anal sphincter repair in rabbit
Source: BMC Gastroenterol. 2021 Dec 15;21:473. doi: 10.1186/s12876-021-02047-2 (PMC8672605; doi:10.1186/s12876-021-02047-2)
Supplement: Supplementary file 1 — Additional file 1. List of antibodies Primer sequences. Table S1: The list of primary and secondary antibodies. Table S2: Primer sequences. [file 12876_2021_2047_MOESM1_ESM.docx]

**Table S1:** The list of primary and secondary antibodies

| **Name** | **Number code** |
| --- | --- |
| Ki67 primary antibody | Thermo Fisher Scientific, MA5-14520 |
| MYH primary antibody | Sigma, M4276 |
| ACTA1 primary antibody | Sigma, A5228 |
| VEGFA primary antibody | Abcam, ab1316 |
| Vimentin primary antibody | Sigma, V6630 |
| Alexa Fluor 594 Goat anti-mouse IgG | Biolegend, 405326 |

**ACTA1:** Skeletal muscle alpha-actin; **MHC:** Myosin heavy chain; **VEGFA:** vascular endothelial growth factor A.

**Table S2:** Primer sequences

| **Ptrimer name** | **Sequence 5´→3´** |
| --- | --- |
| **ACTA1** |  |
| Forward | GATGAAGGAGGGCTGGAAGA |
| Reverse | GACTTCGAGAACGAGATGG |
| **MYH** |  |
| Forward | CTGGCTGGCTGGACAAGAA |
| Reverse | TGAAGAGAGCAGACACGGTC |
| **Vimentin** |  |
| Forward | GACAGGATGTTGACAATGC |
| Reverse | GTCGATCTGGACATGCTGTT |
| **VEGFA** | |
| Forward | TGCTGTAGGAAGCTCATCTC |
| Reverse | GGCTGCTGCAATGATGAAAG |
| **Ki67** |  |
| Forward | AAGGACTGGAAATAGCAGAGG |
| Reverse | GGATGTGATGGCTGATGAA |
| **GAPDH** |  |
| Forward | CTAGAGCAACAGGGTGGTGG |
| Reverse | AGCGTGGTGGGACTGAGTGG |

**ACTA1:** Actin, alpha 1, skeletal muscle; **GAPDH:** Glyceraldehyde-3-phosphate dehydrogenase; **MYH:** Myosin heavy chain ; **VEGFA:** Vascular endothelial growth factor A.
